# After Dinner Rest a While, After Supper Walk a Mile? A Systematic Review with Meta-analysis on the Acute Postprandial Glycemic Response to Exercise Before and After Meal Ingestion in Healthy Subjects and Patients with Impaired Glucose Tolerance

**DOI:** 10.1007/s40279-022-01808-7

**Published:** 2023-01-30

**Authors:** Tobias Engeroff, David A. Groneberg, Jan Wilke

**Affiliations:** 1 Division Health and Performance, Institute of Occupational, Social and Environmental Medicine, Goethe-University Frankfurt, Theodor-Stern-Kai 7, Building 9B, 60590 Frankfurt am Main, Germany; 2 Institute of Occupational, Social and Environmental Medicine, Goethe-University Frankfurt, Frankfurt, Germany

## Abstract

**Background:**

The most effective way to cope with high blood sugar spikes is to engage in physical activity in temporal proximity to food intake. However, so far, it is unclear as to whether there is an optimal time for physical activity around food intake.

**Objectives:**

We aimed to identify the impact of pre- and post-meal exercise on postprandial glucose excursions in humans with and without type 2 diabetes mellitus.

**Methods:**

We conducted a systematic review with meta-analysis, PROSPERO registration number: CRD42022324070. We screened MEDLINE/PubMed, Cochrane/CINAHL/EMBASE, and Web of Knowledge until 1 May, 2022. We used the risk of bias rating with the crossover extension of the Cochrane risk of bias assessment tool II. Standardized mean differences (SMDs, Hedges’ g) with 95% confidence intervals (CIs) were calculated as pooled effect estimates of a random-effects meta-analysis. Eligibility criteria included three-armed randomized controlled trials comparing the acute effects of pre- and post-meal exercise to a no-exercise control in humans.

**Results:**

Eight randomized controlled trials (crossover trials, high risk of bias) with 30 interventions in 116 participants (47 diagnosed with type 2 diabetes, 69 without type 2 diabetes) were eligible. Exercise after meal ingestion (real food or meal replacement drinks) led to a reduction in postprandial glucose excursions compared with exercise before eating (15 effect sizes; SMD = 0.47 [95% CI 0.23, 0.70]) and an inactive control condition (15 effect sizes; SMD = 0.55 [95% CI 0.34, 0.75]. Pre-meal exercise did not lead to significantly lower postprandial glucose compared to an inactive control (15 effect sizes; SMD =  − 0.13 [95% CI − 0.42, 0.17]). The time between meal and exercise (estimate =  − 0.0151; standard error = 0.00473; *Z* =  − 3.19; *p* = 0.001; 95% CI − 0.024, − 0.006) had a moderating influence on postprandial glucose excursions.

**Conclusions:**

Exercise, i.e., walking, has a greater acute beneficial impact on postprandial hyperglycemia when undertaken as soon as possible after a meal rather than after a longer interval or before eating.

**Clinical Trial Registration:**

The review was pre-registered in the PROSPERO database (CRD42022324070). The date of submission was 07.04.2022, with the registration on 08.05.2022.

## Key Points

**Table:** 

Exercise (such as 20 min of walking) has an acute beneficial impact on postprandial hyperglycemia when undertaken as soon as possible after a meal.
Longer intervals between eating and exercising weaken the acute effect on glucose levels.
Exercise prior to a meal does not blunt postprandial hyperglycemia.
Post-meal exercise minimizes glycemic excursions and therefore might lower the risk for low-grade inflammation diseases and cardiovascular diseases.

## Introduction

A large part of the global population accumulates up to 16 h of sedentary time daily and is almost permanently in the fed state due to a frequent intake of food [1]. Because of this postprandial state and inactive setting, macronutrients are consistently entering the blood stream through digestion from where they must be absorbed into other tissues. In view of the limited physical activity (PA)-induced energy expenditure, the metabolism is forced to store large amounts of carbohydrates. Even for healthy humans, the resulting glycemic excursions are a relevant risk factor for low-grade inflammatory (including type 2 diabetes mellitus, non-alcohol fatty liver disease, and rheumatoid arthritis) [2, 3] and cardiovascular diseases [4, 5].

For patients with well-controlled type 2 diabetes, postprandial hyperglycemia is considered to have the largest detrimental impact on long-term glycemic control as indicated by elevated levels of glycated hemoglobin (HbA1c) [6]. Consequently, attenuating meal-induced blood sugar excursions has a major impact on minimizing the risk for these lifestyle-associated diseases and on optimizing the blood sugar management of patients with type 2 diabetes.

The most effective way to cope with high blood sugar spikes is to engage in PA in temporal proximity to food intake [7]. However, so far, it is unclear as to whether there is an optimal time for PA around food intake. In a well-regarded letter, Dr. Chacko, a physician who has diabetes herself, concluded from her personal experience that exercise 30 min to 1 h after meal ingestion might have an optimal effect on postprandial glucose excursions [8]. A few years later, Chacko analyzed the available evidence on effects of PA before a meal, early postprandial (0–29 min after a meal), mid postprandial (30–120 min after a meal), and late postprandial (> 120 min after a meal) systematically and found a superiority of PA in a time window of 30–45 min after food intake [9]. In line with these observations, a narrative review described a larger effect of PA after eating compared with PA before food intake [10]. However, in contrast to the assumptions of Chacko [9], the same research group was able to demonstrate superiority of PA directly after eating compared with activities before or 30 min after eating in healthy participants [11]. Another experimental study [12] even showed the superiority of intensive training in the fasted state. The most recent systematic review [13] concluded that, owing to a lack of suitable studies, it cannot be clearly clarified whether activity in the fasted state or after eating shows a better effect.

Although a beneficial effect of PA can generally be assumed regardless of the meal-exercise timing [7], the available evidence indicates the need for a quantitative evaluation of published studies by means of a meta-analysis. In order to gauge the effect of exercise-meal timing, this study consequently pooled the results of randomized controlled trials comparing pre- and post-meal exercise and an inactive control condition. Furthermore, we aimed to investigate (1) whether the time between meal ingestion and PA influences the effect of postprandial exercise in humans with or without type 2 diabetes and (2) whether exercise duration, type and intensity represent relevant moderators.

## Methods

### Study Design

A systematic review with meta-analysis was performed adhering to the Preferred Reporting Items for Systematic Reviews and Meta-Analyses (PRISMA) guidelines [14]. The review was prospectively registered in the PROSPERO database (CRD42022324070). The date of submission was 07.04.2022, with the registration on 08.05.2022.

### Inclusion and Exclusion Criteria

Studies recruiting participants (over the age of 16 years) with type 2 diabetes were eligible. To be included in the review, the studies had to compare the effects of a pre-meal exercise bout and an intensity-, duration-, and workload-matched post-meal exercise bout, using a randomized controlled design (crossover/parallel group; control/comparator arm: no exercise). The primary outcome of interest was interstitial or blood glucose during the postprandial phase after meal ingestion (real food or meal replacement drinks). Secondary outcomes included data on insulin and fat metabolism based on blood drawings or indirect calorimetry.

### Literature Research

A systematic literature search was performed between February and May 2022 (final search date: 1 May, 2022) Two independent investigators (TE, JW) used standardized syntaxes (see below) to screen the databases PubMed (MEDLINE), Web of Knowledge and the Cochrane Library/Cochrane Central Register of Controlled Trials (CENTRAL, with EMBASE) for eligible articles. In addition, a hand search was conducted in Google Scholar in order to reveal potential gray literature and the reference lists of included articles were checked (cross-referencing). No language restrictions were applied.

Potentially relevant articles were searched adopting the following Boolean search syntax (example for PubMed): (“exercise”[MeSH Terms] OR “sports”[MeSH Terms]) AND (“pre breakfast” OR “pre-breakfast” OR “pre lunch” OR “pre-lunch” OR “pre-dinner” OR “pre dinner” “pre meal” OR premeal OR “pre-meal” OR fasted OR fasting OR postabsorptive OR post-absorptive) AND (“post breakfast” OR “post-breakfast” OR “post lunch” OR “post-lunch” OR “post-dinner” OR “post dinner” “post meal” OR postmeal OR “post-meal” OR “after meal” OR fed OR postprandial OR post-prandial OR postdinner) AND (type 2 diabetes[MeSH Terms] OR diabet*).

Studies identified through the search strategy were screened for between-database duplicates before abstract screening. Subsequently, TE and JW independently screened titles and abstracts of the identified studies to determine whether they met the inclusion criteria. If required, full texts were then assessed to ascertain eligibility for inclusion. Disagreements between investigators were resolved by discussion and, if needed, by consulting a third investigator (DG).

### Data Extraction

Using a standardized extraction form (Excel spreadsheet), we extracted the following descriptive data from the included studies: authors and year of publication, study design, sample size, participant characteristics, interventions, measured outcomes and major findings (outcomes not included in the meta-analysis). One researcher recorded all the pertinent data from the included articles and the other author independently verified the relevance, accuracy and comprehensiveness of the extracted data. Similar to the literature, a consensus process was used to address any disparities and a third reviewer (DG) was asked to address unresolved disagreement. Authors of the studies included in this review who had not reported sufficient details in the published manuscript were personally addressed via e-mail for the provision of further data.

The primary outcome of the meta-analysis was blood glucose during the postprandial phase after meal ingestion. If a study assessed more than one outcome, all data (i.e., means and standard deviations) needed to calculate the effect sizes were extracted. Missing data (means, standard deviations) were imputed from medians, interquartile range, figures and/or confidence intervals using standard procedures [15]. All studies included were screened for common effect estimators to be included in the quantitative analysis.

### Risk of Bias Assessment

Two reviewers (TE and JW) rated the risk of bias of the included studies using the Revised Cochrane risk-of-bias tool (RoB II) extension for randomized crossover trials. The outcomes were graded for risk of bias in each of the following domains: sequence generation, allocation concealment, differences in baseline values, number of participants, period and carryover effects, blinding (participants, personnel and outcome assessment), incomplete outcome data, selective outcome reporting, and other sources of bias. Each item was rated as having a “high risk”, “low risk”, or “unclear risk” of bias. Disagreements were discussed between the raters. Again, if a decision could not be reached after discussion, a third reviewer (DG) was consulted. If applicable, the outcomes’ biases were reported pooled for studies. The risk of bias findings were displayed using a traffic light system as traffic lights and summary plots using robvis [16], an online tool created with the R package robvis. Publication bias was estimated using visual inspection of funnel plots (primary outcome only) created with Jamovi 1.0.7.0 (The Jamovi project, 2021, https://www.jamovi.org; Sydney, NSW, Australia).

### Meta-analysis

Weighted and standardized mean differences (Hedges’ g) were used for data pooling. A restricted maximum-likelihood random-effects meta-analysis model for continuous outcomes was chosen. For variance description, 95% confidence intervals (CIs) were calculated and the summary estimates of the data were displayed using forest plots (mean effect sizes and 95% CIs): (1) overall (main) effects (of pre-meal exercise compared to post-meal exercise) and (2) comparison of pre-meal exercise with a no-exercise control and post-meal exercise with a no-exercise control. For all calculations, exercise group effects were calculated in comparison to each other (pre-meal exercise vs post-meal exercise) and compared to the comparator/control no exercise condition as standardized mean differences. To test for overall effects, *Z*-statistics at a 5% alpha error probability level were calculated for all quantitative comparisons. The moderating influence of exercise intensity, type, and duration as well as the influence of time between meal and post-meal exercise were analyzed in a random-effects model. Heterogeneity between studies was assessed using *I*^2^ and Tau^2^ statistics. All analyses were performed using the MAJOR package of Jamovi (Version 1.0.7.0).

## Results

### Study Selection

**Fig. 1:**
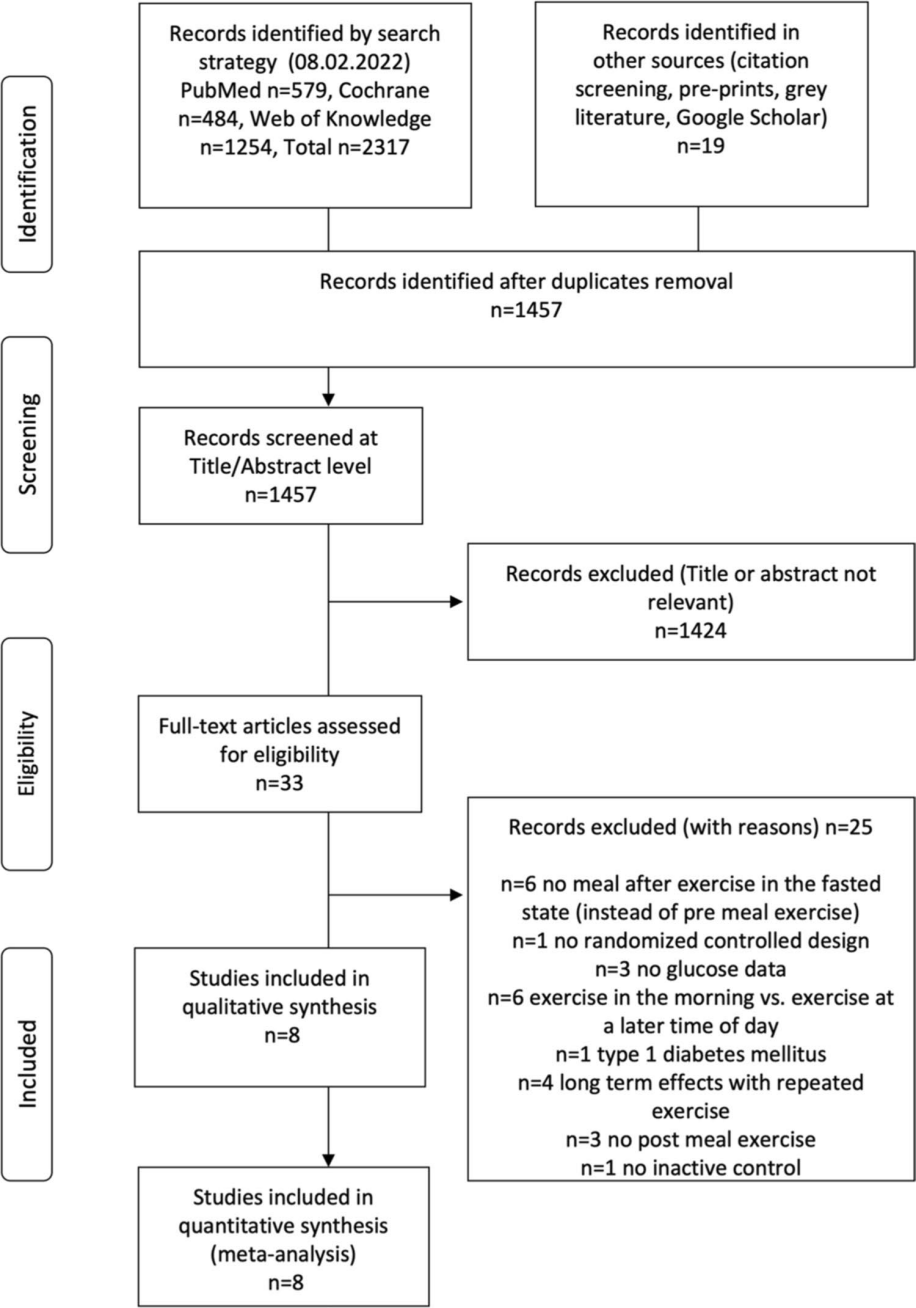
Research, selection, and synthesis of included studies

The initial literature search yielded 1457 unique records. After removing duplicates and applying inclusion and exclusion criteria, eight randomized controlled trials analyzing the acute effects of pre- and post-meal exercise on postprandial glucose were included in the qualitative and quantitative analyses. Figure 1 outlines the research procedure and the flow of the study selection and inclusion.

### Results and Characteristics of Individual Studies

All eight included studies on acute effects were randomized controlled trials with a crossover design and compared the application of one or multiple exercise interventions before and after meal ingestion against a control intervention with an identical meal but without exercise. The results of the individual studies (methodological aspects, participant characteristics), with a focus on the descriptive summary statistics for each group and an overview of secondary outcomes, are displayed in Table 1.

**Table 1:** Study characteristics of the included randomized controlled crossover studies. Information on participants’ characteristics, meal, and exercise intervention protocols as well as descriptive key results and information on secondary outcomes and statistics are displayed

References	Participants, number (*n*) (age in years, height in cm, weight in kg, BMI in kg/m^2^, HbA1c in percent) [mean, SD], additional characteristics, diabetes medication	Meal details [mean, SD]	Exercise details (type, intensity, duration), [mean, SD]	Protocol details (duration between exercise and meal)	Postprandial glucose Mean values, area under the curve (AUC) or incremental area under the curve (iAUC), duration, (number of participants), [mean, SD]	Indirect calorimetry Energy expenditure (EE) in kilojoules (kj) or kilocalories (kcal), heart rate (HR) in beats per minute, Respiratory exchange ratio (RER)	Secondary outcomes	Statistical analysis
Colberg et al. [18]	*n* = 12 (6 female, 6 male) Age = 61.4, 2.7 Height = 171.5, 2.5 Weight = 101.0, 6.9 BMI = 34.5, 2.4 HbA1c = 7.0, 0.3 Type 2 diabetes, oral medication, and diet	Dinner: one of 4 different frozen meals with similar caloric contents (grilled chicken, turkey, stuffed pasta, or breaded fish), 420.9, 4.8 kcal, 43–54 g carbohydrate, 4–10 g fiber, 9–16 g fat, 19–32 g protein	Treadmill walking, moderate intensity (40.3, 3.7% heart rate reserve, self-selected pace perceived as comfortable), 20 min	Exercise beginning 15–20 min post-dinner	11.3, 3.7 mmol/L, iAUC, 240 min, (12)		None	Repeated measures analysis of variance, (rmANOVA)
				Exercise immediately pre-dinner	13.7, 2.2 mmol/L, 240 min, iAUC, (12)			
				Control (dinner)	11.7, 2.0 mmol/L, iAUC, 240 min, (12)			
Heden et al. [17]	*n* = 13 (7 female, 5 male) Age = 48.5, 11.9 Height = 167, 11 Weight = 103.2, 22.8 BMI = 36.7, 5.3 HbA1c = 7.2, 1.1 Type 2 diabetes, oral medication, no diet	Dinner: spaghetti, sauce with beef, garlic bread, lemon-lime flavored soda, 1.5 g of acetaminophen (to assess gastric emptying), 50% carbohydrate, 35% fat, and 15% protein	Resistance exercise (leg press, seated calf raises, seated chest flies, seated back flies, back extensions, shoulder raises, leg curls, and abdominal crunches), vigorous intensity (10 repetition maximum), 46, 1 min	Exercise beginning 45 min post-dinner	638, 487 mmol/L, iAUC, 240 min, (13)	EE = 590, 59 kJ HR = 110, 4 RER = 1.00, 0.01	Metabolic data during exercise: oxygen consumption (no difference), energy expenditure (no difference), RER (no difference), heart rate (higher in post meal exercise), perceived exertion (no difference) Post-meal data: insulin, insulin secretion and C-peptide (lowering effect of post-meal exercise), insulin clearance (lowering effect of pre- and post-meal exercise), glucagon (lowering effect of pre-meal exercise), free fatty acids (increasing effect of pre meal exercise), triacylglycerol and very low-density lipoprotein-1 (lowering effect of post-meal exercise), chylomicrons, very low density lipoprotein-2 (no effects), glucagon-like peptide 1 (lowering effect of post-meal exercise), glucose-dependent insulinotropic peptide (no effect), subjective well-being (increasing effect of pre- and post-meal exercise) Subsequent meal data: no effect on nocturnal or morning glycemic control, fasting glucose, insulin, C-peptide, insulin clearance, insulin resistance, or triacylglycerol	rmANOVA
				Exercise until 20–30 min pre-dinner	746, 371 mmol/L, iAUC, 240 min, (13)	EE = 586, 59 kJ HR = 106, 4 RER = 1.00, 0.01		
				Control (dinner)	909, 325 mmol/L, iAUC, 240 min, (13)	EE = 264, 13 kJ HR n.a RER = 0.83, 0.01		
Nygaard et al. [19]	*n* = 12 (4 female, 8 male) Age = 65, 8 Height = 173, 8 Weight = 73.3, 9.7 BMI = 24.5, 1.9 HbA1c = 6.1, 0.6 Elevated fasting glucose, no oral medication, no diet	Breakfast: 250 mL semi-skimmed milk and cornflakes (1 g carbohydrate per kg bodyweight), 380, 48 kcal, 13, 2 g protein, 3, 1 g fat, 74, 9 g carbohydrate	Treadmill walking, moderate intensity (8% inclination, 12 on a 6–20 perceived exertion scale), 60 min	Exercise beginning 15 min post-breakfast	1477, 105 mmol * 210 min * l^−1^, AUC, (12)		Metabolic data during exercise: heart rate (higher in post-meal exercise), aerobic metabolism (no differences), carbohydrate oxidation (higher in post-meal exercise), fat oxidation (lower in post-meal exercise), blood lactate (higher in post-meal exercise), perceived exertion (higher in post-meal exercise) Subsequent meal data: no effect on postprandial glucose values after the second or third meal after exercise. Significant effect on postprandial glucose after the fourth meal (11 h after breakfast) [post-meal < pre-meal], significant effect on the 10 highest glucose values during the day (post-meal < control, post-meal < pre-meal)	Linear mixed-model analysis
				Exercise until 30 min pre-breakfast	1549, 188 mmol * 210 min * l^−1^, AUC, (12)			
				Control (breakfast)	1576, 199 mmol * 210 min * l^−1^, AUC, (12)			
Terada et al. [12]	*n* = 10 (2 female, 8 male) Age = 60, 6 Height = 172.4, 9.4 Weight = 91.4, 17.1 BMI = 30.8, 5.4 HbA1c = 7.1, 1.0 Type 2 diabetes, oral medication	Breakfast: healthy breakfast, 600, 57 kcal, 50% carbohydrate, 20% protein, 30% fat	Treadmill walking, moderate intensity (55% VO_2peak_), 60 min	Exercise beginning 60 min post-breakfast	240, 183 mmol * 120 min * l^−1^, AUC, (8)	EE = 362, 101 kcal RER = 0.85, 0.03	Metabolic data during exercise: oxygen uptake relative to maximal uptake (%VO_2peak_, no difference), oxygen uptake (no difference), RER (sign higher during post-meal exercise), energy expenditure (no difference)	Linear mixed-model analysis
				Exercise until 60 min pre-breakfast	198, 189 mmol * 120 min * l^−1^, AUC, (9)	EE = 351, 88 kcal RER = 0.82, 0.04		
				Control (breakfast)	355, 66 mmol * 120 min * l^−1^, AUC, (10)			
			Treadmill walking, vigorous intensity (15 intervals each 1 min 100% VO_2peak_ + 4 min 40% VO_2peak_), 60 min	Exercise beginning 60 min post-breakfast	314, 188 mmol * 120 min * l^−1^, AUC, (9)	EE = 371, 94 kcal RER = 0.88, 0.03		
				Exercise until 60 min pre-breakfast	267, 184 mmol * 120 min * l^−1^, AUC, (10)	EE = 354, 85 kcal RER = 0.83, 0.04		
				Control (breakfast)	355, 66 mmol * 120 min * l^−1^, AUC, (10)			
Yunarti et al. [21]	*n* = 10 (10 female) Age = 40.41, 3.09 Height = 161.40, 3.56 Weight = 86.67, 7.26 BMI = 33.22, 2.20 Obesity	Breakfast: no information available	Treadmill walking, moderate intensity, 30 min	Exercise post-breakfast, interval between meal and exercise not indicated	200.2, 4.5 mg/dL, 120 min, (10)		None	n.a
				Exercise pre-breakfast, interval between meal and exercise not indicated	207.6, 4.5 mg/dL, 120 min, (10)			
				Control (breakfast)	211.3, 4.3 mg/dL, 120 min, (10)			
Yoko et al. [20]	*n* = 11 (6 female, 5 male) Age = 42.7, 9.4 Height = 164.8, 5.3 Weight = 62.9, 10.9 BMI = 23.1, 4.1	Lunch: 100 g rice and ad libitum intake of protein and fat, ≥ 40 g carbohydrate, no information on the amount of protein and fat intake	Walking, moderate intensity (2 min 4 km/h and 18 min 6 km/h), 20 min	Exercise beginning 20 min post-lunch	2143, 1245 mg * 120 min * dL^−1^, iAUC, (11)		None	Wilcoxon signed-rank test
				Exercise immediately pre lunch	3050, 1654 mg * 120 min * dL^−1^, iAUC, (11)			
				Control (lunch) for post-lunch exercise	3393, 1781 mg * 120 min * dL^−1^, iAUC, (11)			
				Control (lunch) for pre-lunch exercise	3599, 2270 mg * 120 min * dL^−1^, iAUC, (11)			
			Resistance exercise (pushups, squats, front bridges), moderate intensity (18 min resistance exercise and 2 min stretching), 20 min	Exercise beginning 20 min post-lunch	2520, 1659 mg * 120 min * dL^−1^, iAUC, (9)			
				Exercise immediately pre-lunch	3230, 2214 mg * 120 min * dL^−1^, iAUC, (9)			
				Control (lunch) for post-lunch exercise	3058, 2283 mg * 120 min * dL^−1^, iAUC, (9)			
				Control (lunch) for pre-lunch exercise	3498, 1964 mg * 120 min * dL^−1^, iAUC, (9)			
Solomon et al. [11]	*n* = 16 (5 female, 11 male) Age = 31, 11 Weight = 72.3, 10.6 BMI = 23.5, 2.8 HbA1c = 5.3, 0.3	Breakfast: meal replacement drink (Nurishment; Dunns River Brands, Frisco, TX, USA), 500 kcal, 71 g carbohydrate (57% of total kcal), 13 g fat (26% of total kcal), 24 g protein (19% of total kcal)	Standing, light intensity (rating of perceived exertion 6, 1 on a 6–10 scale), 30 min	Exercise (standing) immediately post-breakfast	663, 63 mg * 120 min * dL^−1^, AUC, (16)	EE = 54, 14 kcal RER = 0.87, 0.10		rmANOVA
				Exercise (standing) beginning 30 min post-breakfast	683, 51 mg * 120 min * dL^−1^, AUC, (16)			
				Exercise (standing) immediately pre-breakfast	714, 66 mg * 120 min * dL^−1^, AUC, (16)			
				Control (breakfast)	698, 36 mg * 120 min * dL^−1^, AUC, (16)			
	*n* = 16 (5 female, 11 male) Age = 31, 11 Weight = 72.3, 10.6 BMI = 23.5, 2.8 HbA1c = 5.3, 0.3		Treadmill walking, light-to-moderate intensity (3.5, 0.5 miles per hour, rating of perceived exertion 11, 2 on a 6–20 scale), 30 min	Exercise (walking) immediately post-breakfast	617, 58 mg * 120 min * dL^−1^, AUC, (16)	EE = 123, 19 kcal RER = 0.87, 0.06		
				Exercise (walking) beginning 30 min post-breakfast	669, 84 mg * 120 min * dL^−1^, AUC, (16)			
				Exercise (walking) immediately pre-breakfast	675, 686 mg * 120 min * dL^−1^, AUC, (16)			
				Control (breakfast)	673, 96 mg * 120 min * dL^−1^, AUC, (16)			
	*n* = 16 (11 female, 5 male) Age = 24, 7 Weight = 66.0, 11.3 BMI = 23.0, 3.4 HbA1c = 5.1, 0.4		Resistance exercise with bodyweight (3 times: 10 squats, 10 push-ups, 10 sit-ups, 20 alternate leg forward lunges), light-to-moderate intensity (rating of perceived exertion 11, 2 on a 6–20 scale), 7.1, 1.8 min	Exercise (resistance) immediately post-breakfast	624, 57 mg * 120 min * dL^−1^, AUC, (16)	EE = 53, 19 kcal RER = 1.04, 0.09		
				Exercise (resistance) beginning 30 min post-breakfast	655, 99 mg * 120 min * dL^−1^, AUC, (16)			
				Exercise (resistance) immediately pre-breakfast	663, 68 mg * 120 min * dL^−1^, AUC, (16)			
				Control (breakfast)	673, 51 mg * 120 min * dL^−1^, AUC, (16)			
Derave et al. [22]	*n* = 7 (male) Age = 45, 11 Weight = 108, 14 BMI = 34, 3 Metabolic syndrome (3 or more of the following: waist circumference > 102 cm, blood pressure > 130/85 mmHg, serum triglycerides > 150 mg/dL, high-density lipoprotein cholesterol < 40 mg/dL, fasting glucose > 110 mg/dL but < 126 mg/dL, or fasting insulin > 15 uU/mL)	Breakfast: 20 kJ/kg body weight, 82% carbohydrate, 9% protein, 9% fat	Bicycle ergometer, moderate intensity (60% VO_2max_), 45 min	Exercise immediately post-breakfast	2970, 1090 mg * min * dL^−1^, iAUC, (7)	HR = 138, 17 RER = 0.91, 0.01	Metabolic data during exercise: oxygen uptake (post-meal < pre-meal), carbon dioxide production (post-meal > pre-meal), RER (post-meal > pre-meal), carbohydrate oxidation rate (post-meal > pre-meal), fat oxidation rate (post-meal < pre-meal), HR (no effect) Post-meal data: insulin (no effect), triglycerides (no effect) Subsequent meal data: no effect on postprandial glucose, insulin or triglyceride values after the second or third meal after exercise	rmANOVA
				Exercise immediately pre-breakfast	5230, 2690 mg * minutes * dL^−1^, iAUC, (7)	HR = 133, 22 RER = 0.87, 0.03		
				Control (breakfast)	3740, 1020 mg * min * dL^−1^, iAUC, (7)			

*BMI* body mass index, *HbA1c* glycated hemoglobin, *n.a. *not available, *SD* standard deviation

Overall, the study sample comprised 116 participants, 40 of whom were female. Four studies included an overall number of 47 patients with type 2 diabetes and assessed the effects of various activities including 46 min of resistance exercise [17], treadmill walking for 20 min with moderate intensity [18], and treadmill walking for 60 min with moderate [12, 19] or high intensity [12]. One study of participants with type 2 diabetes compared moderate-intensity and vigorous-intensity exercise and reported a higher impact of vigorous intensity exercise on interstitial glucose outcomes [12]. The time intervals between exercise and meal ingestion varied between 0 and 60 min for exercise prior to meal ingestion and between 15 and 60 min for exercise after meal ingestion. Additionally, the time of meal ingestion (breakfast, lunch or dinner) and the type of meal varied across studies. Two studies on participants with type 2 diabetes assessed the impact of exercise around dinner [17, 18] and two around breakfast [12, 19]. In one study, breakfast included milk and cornflakes [19] while in the other study, a meal replacement drink was ingested [12]. Dinner was either an Italian pasta-based meal [17] or participants were able to choose between four different meals with matched caloric content [18].Three studies included a total number of 69 participants without type 2 diabetes and analyzed the effect of moderate-intensity treadmill walking for 20 [20] and 30 min [11, 21], the effect of bodyweight resistance exercise for 20 min [20] and 7 min [11], and the effect of standing upright for 30 min [11]. Two studies compared the effect of resistance exercise and walking [11, 20] and one of these studies reported a greater impact of walking compared with bodyweight resistance exercise [20]. Again, the time intervals between meal and exercise varied between studies. Although all studies on healthy participants [11, 20, 21] applied designs in which exercise before meal ingestions ended immediately before eating, the time interval between meal ingestion and exercise in the postprandial state varied between 0 and 30 min. Only one of the included studies analyzed the impact of exercise-meal timing in healthy subjects and detected a greater impact of exercise immediately after meal ingestion on postprandial glucose compared with the effect of exercise 30 min after meal ingestion [11]. Two studies on healthy participants assessed the impact of exercise around breakfast, which consisted of either a replacement drink [11] or a normal breakfast [21], and one around lunch (100 g of rice combined with an ad libitum amount of side dishes) [20].

### Blood and Interstitial Glucose

**Fig. 2:**
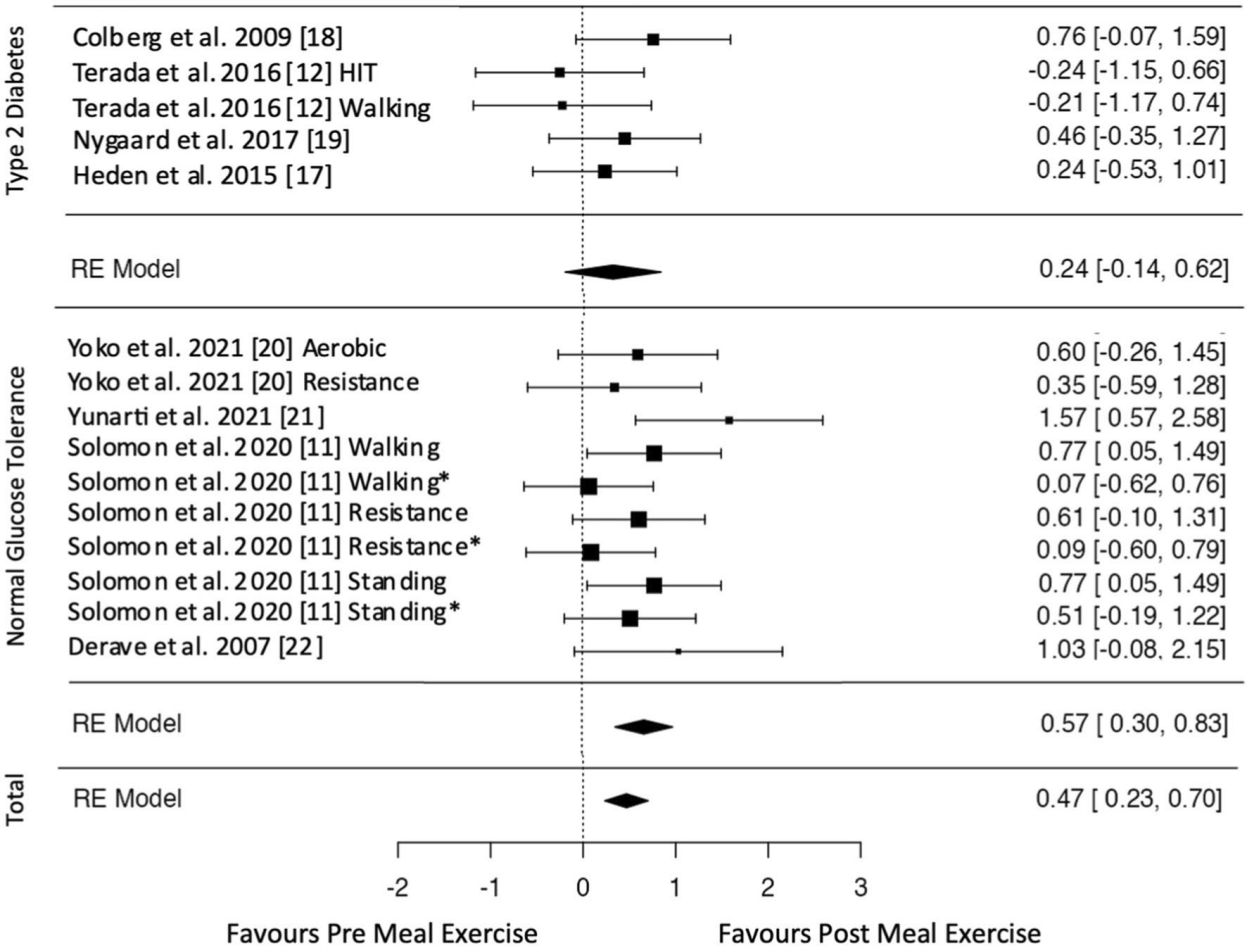
Pooled effect size estimates (standardized mean differences and confidence intervals) for postprandial glucose. Overall effects for post-meal exercise in comparison to pre-meal exercise and effects for subgroups with and without type 2 diabetes mellitus are displayed. *HIT* high-intensity interval, * indicates that exercise is delayed by 30 min after a meal

**Fig. 3:**
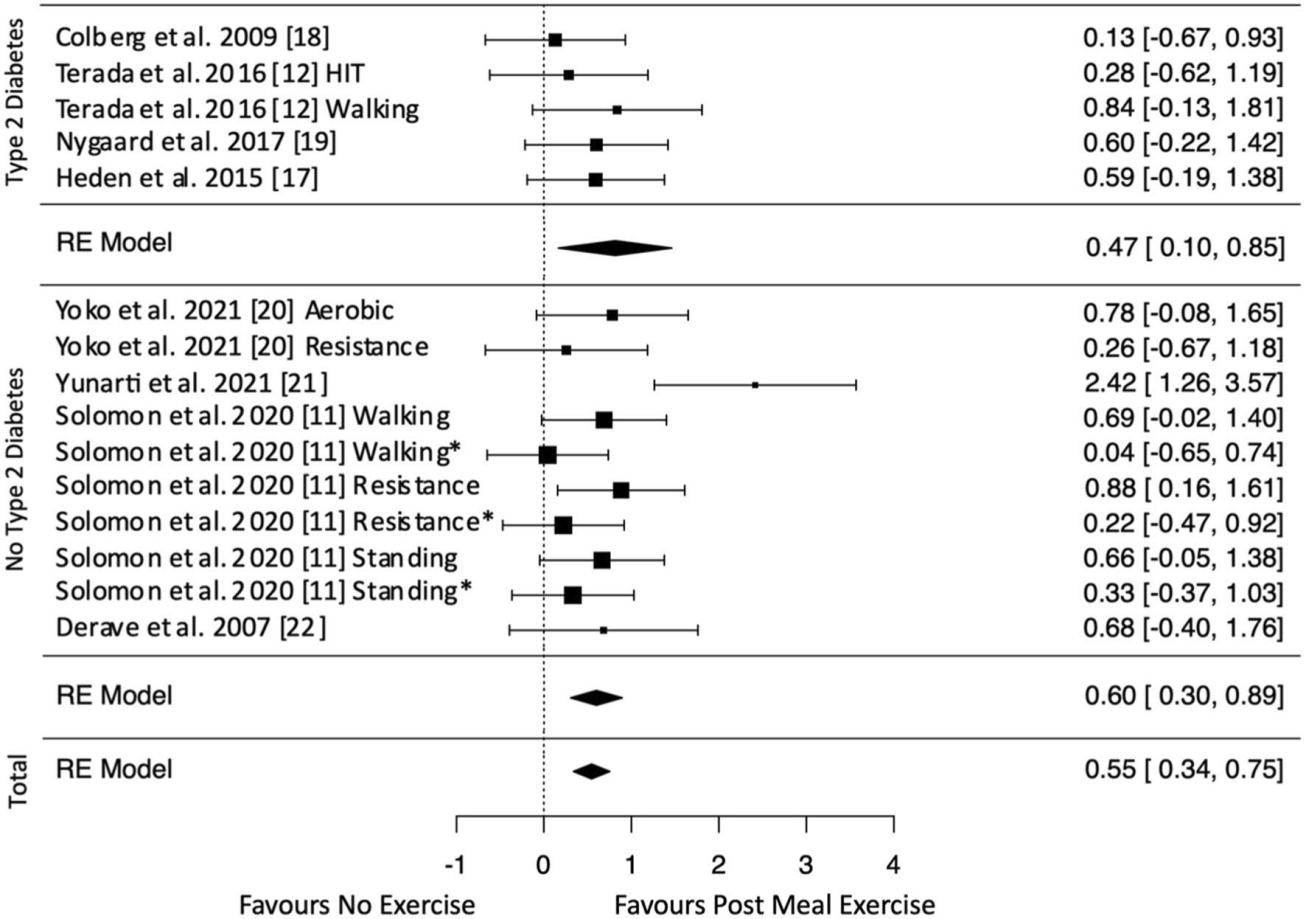
Pooled effect size estimates (standardized mean differences and confidence intervals) for postprandial glucose. Overall effects for post-meal exercise in comparison to a no-exercise control and effects for subgroups with and without type 2 diabetes mellitus are displayed. *HIT* high-intensity interval, * indicates that exercise is delayed by 30 min after a meal

**Fig. 4:**
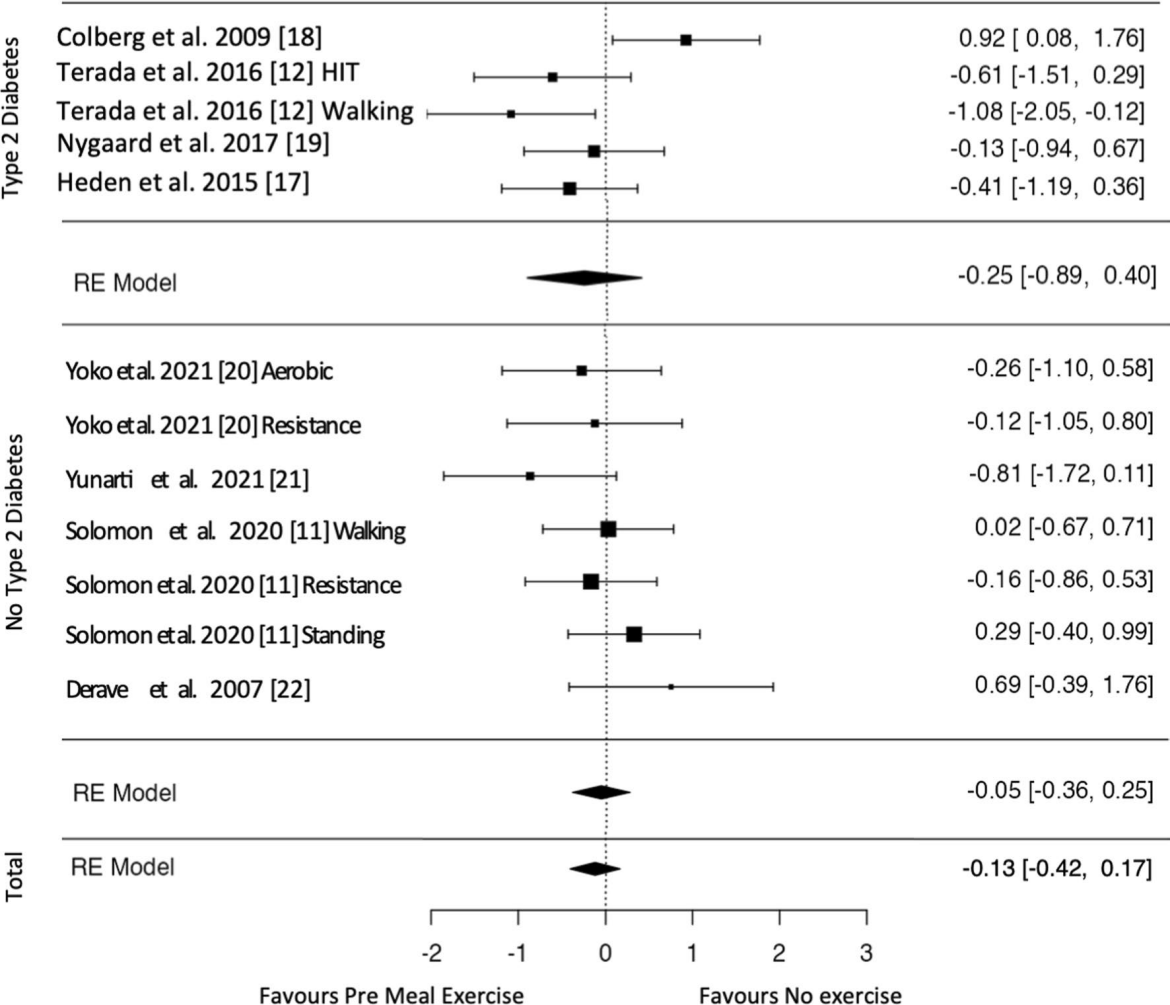
Pooled effect size estimates (standardized mean differences and confidence intervals) for postprandial glucose. Overall effects for pre-meal exercise in comparison to a no-exercise control and effects for subgroups with and without type 2 diabetes mellitus are displayed. *HIT* high-intensity interval

All included studies assessed the postprandial glycemic response either via interstitial glucose [11, 12, 19] or blood glucose [17, 18, 20, 21] measurements. The effect estimates for glucose data are displayed in Figs. 2, 3, 4. Exercise after meal ingestion led to a greater reduction in postprandial glucose excursions than exercise before eating (Fig. 2; 15 effect sizes; standardized mean difference [SMD] = 0.47 [95% CI 0.23, 0.70]). On the subgroup level, this finding was confirmed for participants without type 2 diabetes (ten effect sizes; SMD = 0.57 [95% CI 0.30, 0.83]). The meta-analysis on participants with type 2 diabetes did not reach statistical significance (five effect sizes; SMD = 0.24 [95% CI − 0.14, 0.62]).

Overall, post-meal exercise induced decreased postprandial glucose levels compared with the inactive control (Fig. 3; 15 effect sizes; SMD = 0.55 [95% CI 0.34, 0.75]). A subgroup analysis showed glucose-lowering effects for participants with (five effect sizes; SMD = 0.47 [95% CI 0.10, 0.85]) and without type 2 diabetes (ten effect sizes; SMD = 0.60 [95% CI 0.30, 0.89]). Pre-meal exercise effects on postprandial glucose did not reach statistical significance either overall (15 effect sizes; SMD =  − 0.13 [95% CI − 0.42, 0.17]) or in the subgroup analyses of participants with (five effect sizes; SMD =  − 0.25 [95% CI − 0.89, 0.40]) and without type 2 diabetes (seven effect sizes; SMD =  − 0.05 [95% CI − 0.36, 0.25]) [Fig. 4 shows overall and subgroup analyses].

Analysis of potential moderators revealed a significant influence of the time elapsed between meal and exercise, indicating a greater effect of activities immediately after finishing a meal compared with activities with a delay of up to 60 min after finishing a meal (estimate =  − 0.0151; standard error = 0.00473; *Z* =  − 3.19; *p* = 0.001; 95% CI − 0.024, − 0.006). Duration, type, and intensity of exercise as well as the type of glucose drawing (blood and interstitial) did not influence the effect on postprandial glucose (*p* > 0.05).

Two studies investigated the sustained effects of exercise on postprandial metabolism after the second, third [22], and fourth meal after exercise [19] and detected a delayed beneficial effect of post-meal exercise on postprandial glucose after the fourth meal of a day [19]. Furthermore, Nygaard and colleagues were able to show a beneficial effect of post-meal exercise on the ten highest glucose values during the day [19]. One study assessed nocturnal and morning glycemic control and fasting glucose values on the next day (after exercise pre- and post-dinner) but found no significant effects on these outcomes [17].

### Secondary Post-meal Metabolic Outcomes

Findings for secondary outcomes are indicated in Table 1. Although most studies sampled glucose data at additional timepoints (during the postprandial phase after the second and third meal after exercise) or analyzed additional measures (such as maximal values or glycemic variability), only one study [17] evaluated additional metabolic outcomes such as measures of fat metabolism and hormones (detailed results in Table 1). The authors reported a beneficial effect of post-meal exercise on blood triacylglycerol. This study, furthermore, analyzed insulin data and reported lower postprandial insulin secretion after post-meal exercise compared with pre-meal exercise but comparable effects of both exercise types on insulin clearance compared to a control intervention [17]. These outcomes were not included in the meta-analysis because of the small number of studies involved.

### Metabolism During Exercise

Indirect calorimetry was applied by five studies which reported respiratory exchange ratio values between 0.83 and 1.00 [11, 12, 17, 19, 22]. The energy expenditure during exercise varied between 54 and 371 kcal [11, 12, 17]. Two studies detected higher heart rate values during post-meal exercise compared with pre-meal exercise [17, 19]. Two studies reported higher respiratory exchange ratio values [12, 22], two studies detected higher carbohydrate oxidation values [19, 22], and one study found higher lactate values [19] during post-meal exercise in participants without type 2 diabetes. In contrast, respiratory exchange ratio values were comparable during pre- and post-meal exercise in people with type 2 diabetes in one study [17]. Four studies analyzed oxygen uptake, substrate utilization, and energy expenditure [12, 17, 19, 22]. Whereas three studies reported no differences between exercise conditions [12, 17, 19], one study found lower oxygen uptake and fat oxidation but higher carbohydrate oxidation during exercise post-meal compared with exercise prior to meal consumption. These outcomes were not included in the meta-analysis because of the small number of studies involved.

### Risk of Bias and Publication Bias

**Fig. 5:**
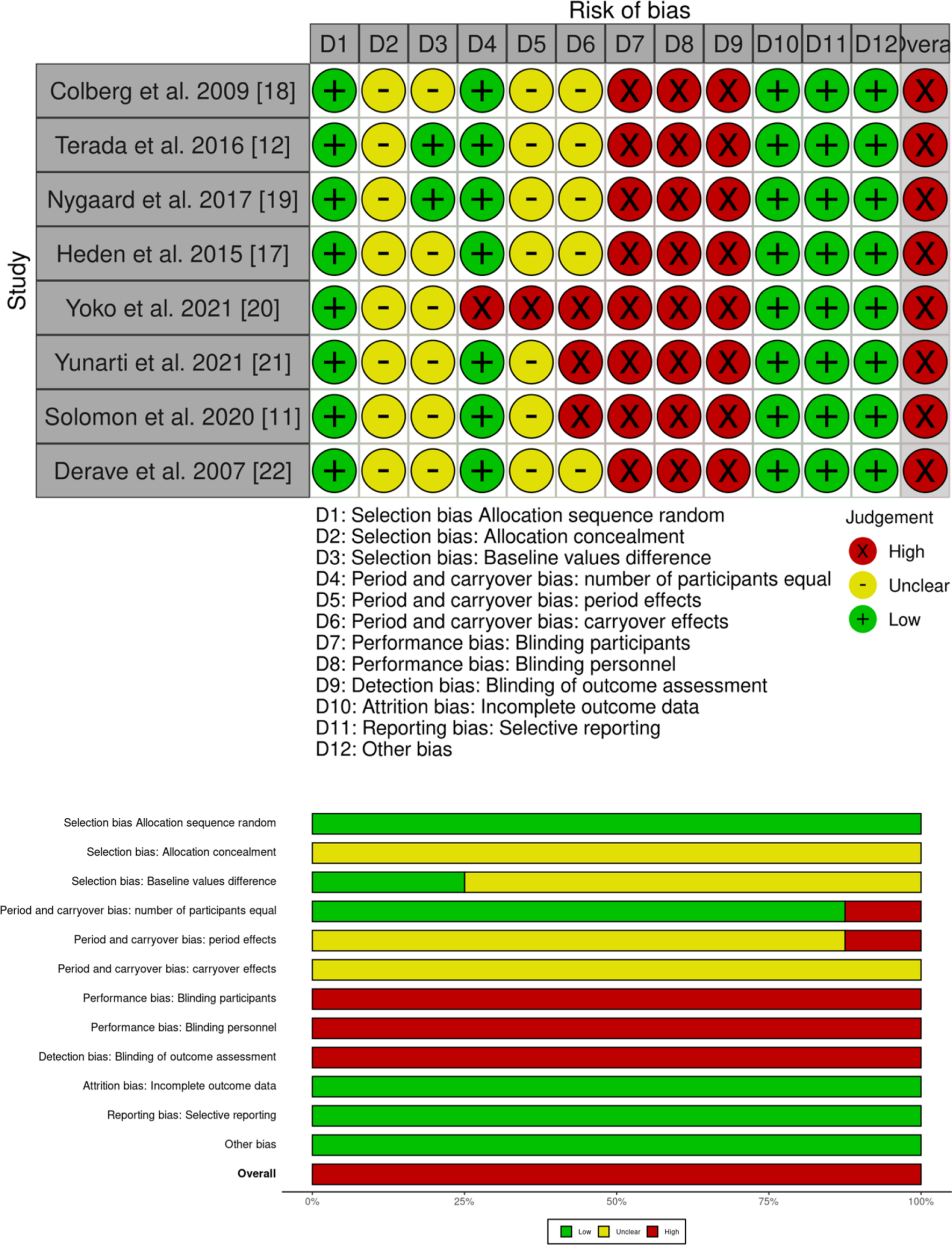
Risk of bias rating for each item, displayed as traffic light plots (above) and as summary bar plots (below). The colors indicate high (red), unclear (yellow), or low (green) risk for the respective bias domain/item

**Fig. 6:**
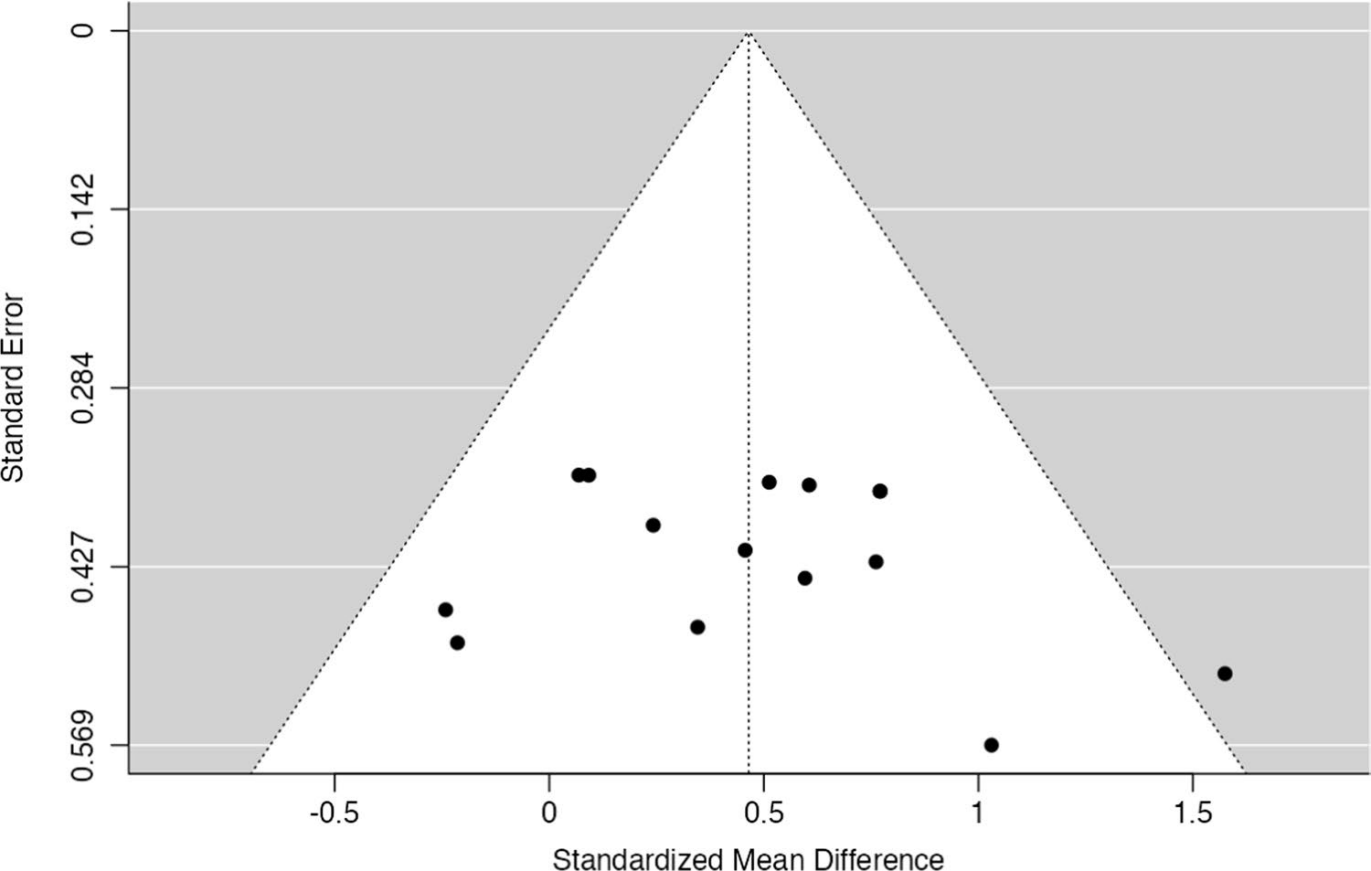
Publication bias visualized as funnel plot of all included studies. Each standardized mean difference (SMD) and their corresponding standard error (SE) for the comparison of pre-meal and post-meal exercise effects on postprandial glucose are plotted

The included studies showed a high overall risk of bias. Detailed ratings for the risk of bias on the study/outcome level are displayed in Fig. 5. Funnel plots for the estimation of publication bias are shown in Fig. 6. Visual inspection revealed a mostly symmetrical distribution of effect sizes and an overall low risk of publication bias.

## Discussion

This systematic review with meta-analysis was the first to directly compare the effects of pre-meal and post-meal exercise to a no-exercise control by including only three-armed study designs. We found evidence that exercise after meal ingestion is feasible for lowering postprandial glucose excursions in humans with and without type 2 diabetes compared with a no exercise control. Our data, furthermore, show that post-meal exercise is more beneficial to cope with postprandial glucose excursions than pre-meal exercise. Exercise prior to a meal ingestion showed no significant effect on postprandial blood glucose compared to an inactive control.

Engaging in PA during the postprandial metabolic state after meal ingestion seems to be the most successful approach to attenuate meal-induced blood sugar excursions. The optimal time point to get active is as soon as possible in the early postprandial phase (0–29 min after meal). Despite the limited number of effect sizes, a trend for lower effects of mid-postprandial activity (30–120 min after meal) was found. The study with the longest duration between eating and activity (60 min) even showed a trend for better effects of walking prior to meal ingestion on postprandial hyperglycemia in patients with type 2 diabetes [12]. One study, to date, has compared activities in the late postprandial state (> 120 min after meal) and reported beneficial effects of exercise at a time interval of up to 5 h from eating [23], but based on the available evidence a superior effect compared to exercise closer to eating seems unlikely. Overall, these findings on exercise timing indicate that insulin independent clearance of glucose from the bloodstream might be most efficient immediately after oral glucose uptake [24]. Although insulin-independent GLUT-4 glucose transporter expression on the muscle-cell membrane could also increase glucose uptake following activities which induced intracellular glycogen replenishment, other mechanisms such as increased enzyme linked substrate metabolization and skeletal muscle blood flow are more likely to affect glucose spikes just whilst they are appearing [24]. Additionally, exercise-induced mechanisms, such as excess post-exercise oxygen consumption, replenishment of phosphagen system, and lactate processing [25], could lead to larger effects in the postprandial state with excess glucose availability. One of the included studies reported that multiple short exercise bouts during the postprandial state seem to be more effective than a duration-matched single bout of pre-meal or post-meal exercise in regulating hyperglycemia [21]. Comparable results can be found in a study comparing the effect of exercise in the fasted state to multiple exercise breaks after meal ingestion [26]. This study could additionally confirm an influence of the calorie amount of a self-selected breakfast on the postprandial glucose response and the effect of post-meal exercise [26]. Because of the different types and standardizations of the amount of food in the included studies, we could not systematically analyze the influence of factors such as caloric content, food type, or macronutrient composition in our meta-analysis. There is clear evidence that exercise intensity and duration have an impact on the glucose-lowering effect of exercise in people with type 2 diabetes [23, 27, 28]. However, duration, type, and intensity of exercise did not influence the differences between the effects of pre- meal and post-meal exercise on postprandial glucose in our moderator analysis. The interpretation of our results must take into account the small number of studies and the narrow range of intensities, with only one study of light PA (standing) [11], applied in different types of activity. Overall, more evidence is needed concerning the interaction and influence of the meal taken and the exercise session performed.

Some of the included studies allow further insights into metabolic differences between pre- and post-meal exercise based on indirect calorimetry and blood-based assessments. Respiratory exchange ratios indicate that the energy sources during both exercise types were a combination of fatty acids and glucose [11, 12, 17, 19, 22]. A direct comparison of pre- and post-meal exercise shows higher respiratory exchange ratio [12, 22], carbohydrate oxidation [19, 22], and lactate values [19] during post-meal exercise. In line with these findings, an early study by Schneiter and colleagues already detected increased carbohydrate use but decreased fat turnover if exercise was applied after meal ingestion compared with pre-meal exercise [29]. Based on the combination of indirect calorimetry and labeled glucose ([^13^C]glucose), this group further showed that exogenous glucose was the main source of energy during exercise in the postprandial state whereas muscle glycogen (65%) and lipids (35%) were used during exercise in the fasted state [29]. These results suggest that beneficial effects of post-meal exercise might be induced by a shift in substrate utilization and the insulin-independent use of the glucose directly after ingestion.

Contrary to the ongoing trend towards exercise in the fasted state and the assumption that this approach might lead to lower blood insulin and beneficial changes in lipid metabolism [30], the postprandial glucose values obtained in this meta-analysis point towards the opposite direction. One of the included studies analyzed glycemic response and additional metabolic outcomes and reported a larger favorable effect of post-meal exercise on insulin secretion and blood triacylglycerol levels [17]. Contrary to these findings, a study of the effects of pre-meal exercise compared to multiple post-meal exercise bouts reported favorable effects on postprandial lipoprotein metabolism only for pre-meal exercise [31]. To explain the discrepancies between research on fasted exercise [30] and our data on pre- and post-meal exercise, differences in study design and especially in dietary stimuli have to be considered. A number of studies included in the review by Hansen et al. [30] included a standardized meal only in the study arm with postprandial exercise. Consequently, these studies compared the metabolic response to exercise in the fasted state over a standardized timeframe (during which the participants continued fasting) with the response to exercise after meal ingestion over an identical amount of time in the postprandial state. Using this approach, the caloric intake and therefore the metabolic stimulus during trial arms are not comparable. Consequently, further studies are needed to evaluate the effects of exercise before and after meal ingestion on insulin and fat metabolism.

As only eight studies with 15 effect sizes were available to be analyzed following our rigorous inclusion and exclusion criteria, the number of included studies was quite small. Moreover, the studies included showed a high individual risk of bias. Conversely, publication bias seemed to be low. Based on the small number of studies, the descriptive data, the funnel plot, and the rating, we found no clear indicators for problems relating to heterogeneity, sample size, or publication bias. Consequently, we performed no sensitivity analysis. On the subgroup level, our main finding concerning the direct comparison between pre-meal and post-meal exercise was only confirmed for healthy participants. The meta-analysis on participants with type 2 diabetes included only five effect sizes out of four studies and showed a significant effect of post-meal exercise compared with a no-exercise control (and no effect of pre-meal exercise compared to a control) but only a tendency to beneficial effects of post-meal exercise compared to pre-meal exercise. Although the lack of statistical significance could at least partially be explained by the small number of studies and the long delay of up to 60 min between meal ingestion and exercise (meal exercise timing) in two of the five included trials [12], there are some metabolic factors that need to be considered as possible alternative explanations. Aside from lower insulin sensitivity in various tissues including skeletal muscle cells and higher pancreatic insulin release, type 2 diabetes is also linked to mitochondrial dysfunction and decreased oxidative enzyme capacity, which could lead to a lower rate of glucose turnover during exercise even with comparable relative intensity [32]. Another explanation for the less pronounced effects compared with healthy participants could be that most of the included participants with type 2 diabetes were receiving oral medication such as metformin, which is reported to inhibit complex I of the mitochondrial electron transport chain [33] and blunt the exercise-related upregulation of AMP-activated protein kinase. Earlier studies reported evidence that these interactions might constrain exercise effects [34, 35]. Furthermore, patients with diabetes are likely to be unaccustomed to physical exercise and therefore could have experienced a higher release of stress hormones including norepinephrine and epinephrine [18, 36]. One experimental trial reported that such a sympathoadrenal reaction is even higher after postprandial exercise compared with exercise in the fasted state [37]. As catecholamines have a glucose-raising effect, they could limit the glucose-lowering effect of exercise in patients with diabetes overall and lead to a lesser advantage of post-meal activities. So far, only one study has compared the effects of pre- and post-meal exercise in patients with type 1 diabetes and reported a greater beneficial impact of post-meal exercise on glucose excursions [38].

Regarding the practical relevance of our results, in addition to the acute effects, the medium- and long-term effects of exercise on metabolism must also be considered. Three of the included studies [17, 19, 22] investigated sustained effects of a single bout of exercise. Only one detected an acute influence on postprandial glucose after the fourth meal following post-meal exercise [19]. No effects on other subsequent meals [22] or on nocturnal and morning glycemic control and fasting glucose values on the next day (after exercise around dinner) [17] were found. These data indicate that activities such as walking for 20 min should be either undertaken after all meals or at least after meals in a sedentary setting such as office jobs or in the evening.

A study of post-meal activity over a period of 2 weeks indicates that the effect on postprandial hyperglycemia in patients with type 2 diabetes is repeatable and has the highest impact on evening meals [39]. In line with the assumption that postprandial hyperglycemia has the largest detrimental impact on elevated levels of HbA1c [6], long-term studies with timeframes that range from 8 [40] to 12 weeks [41] confirmed better effects for regular post-meal compared with pre-meal training on HbA1c. In contrast, one study reported beneficial effects of both exercise before and after meal ingestion on various metabolic outcomes including HbA1c but was not able to confirm a superior effect of post-meal exercise with moderate intensity which was completed in the mid-postprandial phase  1–2 h after the meal [42]. Another study was not able to show beneficial effects on glycemic variables when regular postprandial PA was performed at only light-intensity over a timeframe of 12 weeks [43]. Overall, this research indicates that regular postprandial exercise with moderate intensity shortly after meal consumption is likely to induce beneficial long-term effects. However, based on the contradictory results, future studies are necessary to further analyze the impact of exercise prerequisites such as intensity and timing.

## Conclusions

Exercise (such as 20 min of walking) has an acute beneficial impact on postprandial hyperglycemia when undertaken as soon as possible after a meal. Longer intervals between eating and exercising weaken the effect on glucose levels. Exercise prior to a meal does not blunt postprandial hyperglycemia. This effect seems especially relevant in sedentary settings during working hours or in the evening in which macronutrients are provided consistently through digestion and the metabolism would be otherwise forced to store large amounts of carbohydrates. Our data suggest that post-meal exercise minimizes glycemic excursions in healthy humans and patients with type 2 diabetes. Although the extrapolation of the long-term effects based on our data is speculative, earlier studies already suggest that decreased postprandial glycemic load lowers the risk for low-grade inflammation diseases (including type 2 diabetes, non-alcohol fatty liver disease, and rheumatoid arthritis) [2, 3] and cardiovascular diseases [4, 5]. Furthermore, the acute lowering effect of post-meal exercise on the meal-induced hyperglycemia in people with type 2 diabetes might improve long-term glycemic control and reduce the likelihood of further health consequences [18]. In summary, our evidence confirms that the optimal time for PA around food intake is right after the meal. Consequently, the saying should be rephrased to “After dinner walk a while, after supper walk again”.

## Declarations

### Availability of data and material

The data set for meta-analytic calculations will be made available via direct contact with the investigator and after approval of a written proposal and a signed data access agreement.
